# FliC’s Hypervariable D3 Domain Is Required for Robust Anti-Flagellin Primary Antibody Responses

**DOI:** 10.4049/immunohorizons.1800061

**Published:** 2019-09-05

**Authors:** Américo H. López-Yglesias, Chun-Chi Lu, Xiaodan Zhao, Tiffany Chou, Tim VandenBos, Roland K. Strong, Kelly D. Smith

**Affiliations:** * Department of Pathology, University of Washington, Seattle, WA 98195; † Division of Basic Sciences, Fred Hutchinson Cancer Research Center, Seattle, WA 98109

## Abstract

Bacterial flagellin is a well-known agonist of the innate immune system that induces proinflammatory responses through the TLR5 and Naip5/6 recognition pathways. Several clinical trials investigating flagellin fusion proteins have demonstrated promising results for inducing protective immunity toward influenza virus, which has been largely attributed to flagellin’s ability to activate TLR5. Our laboratory previously demonstrated that the *Salmonella enterica* serovar Typhimurium flagellin protein, FliC, induces Ab responses in mice through a third pathway that is independent of TLR5, Casp1/11, and MyD88. In this study, we further define the structural features of FliC that contribute to this unknown third pathway. By destroying the Naip5/6 and TLR5 recognition sites, we demonstrate that neither were required for the TLR5-, inflammasome- and MyD88-independent Ab responses toward FliC. In contrast, deletion of FliC’s D3 or D0/D1 domains eliminated primary anti-flagellin Ab responses. For optimal primary and secondary anti-flagellin Ab responses we show that TLR5, inflammasome recognition, and the D3 domain of FliC are essential for flagellin’s robust immunogenicity. Our data demonstrate that the D3 domain of FliC influences immunogenicity independent of the known innate recognition sites in the D0/D1 domains to augment Ab production. Our results suggest full-length FliC is critical for optimal immunogenicity and Ab responses in flagellin-based vaccines.

## INTRODUCTION

The innate immune system is armed with germline encoded pattern recognition receptors (PRRs) that recognize a multitude of pathogen-associated molecular patterns (PAMPs). The recognition of PAMPs by PRRs leads to the induction of proinflammatory responses, including the activation of mononuclear phagocytes that engulf, process, and present Ags, initiating adaptive immune responses (1). Two sets of PRRs that have been intensively studied are TLRs and NOD-like receptors (NLRs). TLRs recognize various PAMPs, including bacterial lipopeptides (TLR1, 2, 6), glycolipids (TLR4), nucleic acids (TLR3, 7, 8, 9), and proteins (TLR5, TLR11, and TLR12) (1, 2).

TLR5 recognizes flagellin and is expressed by epithelial cells, neutrophils, monocytes, and dendritic cells (3–5). TLR5 signaling is dependent on the adaptor protein MyD88 that is essential for downstream signaling via NF-κB and the MAPK pathways (6, 7). TLR5 recognition of flagellin induces cytokine and chemokine production that is MyD88 dependent (8–10). Several studies support the requirement for TLR5 signaling via MyD88 to induce T cell-dependent Ab responses toward flagellin (10–13). However, recent studies have also shown that TLR5 enhances anti-flagellin T cell responses in a MyD88-independent manner, suggesting that TLR5 also functions as an endocytic receptor to enhance flagellin processing and presentation by dendritic cells (13, 14).

NLRs are another set of PRRs that play an essential role in detecting various PAMPs (15). NLRs are a key group of cytosolic sensors that detect both PAMPs and endogenous danger signals. One set of distinct NLRs is the Naip family. In mice, there are at least four functional Naips: Naip1, Naip2, Naip5, and Naip6, which recognize needle, rod, or flagellin proteins from various bacteria (16, 17). Naip5 and Naip6 (Naip5/6) recognize cytosolic flagellin and activate the inflammasome through recruitment of Nlrc4, which triggers caspase 1 (Casp1) (18, 19). Activation of the Naip5/6 inflammasome leads to the secretion of bioactive forms of IL-18 and IL-1β, calcium-dependent secretion of eicosanoids, and cell death, termed pyroptosis (19).

TLR5 and the inflammasome play redundant roles in IgG1 anti-FliC responses following FliC immunizations, and elimination of both pathways significantly reduces IgG1 anti-FliC Abs (8, 20). In contrast, TLR5 and the inflammasome play nonredundant roles for IgG2c anti-FliC responses; elimination of either flagellin recognition pathway significantly reduces IgG2c anti-FliC Ab responses and elimination of both pathways virtually eliminates IgG2c anti-flagellin responses in mice (8). IgA anti-FliC Ab responses are TLR5 and MyD88 dependent but inflammasome independent (8, 21). Hence, TLR5 and the inflammasome work together to promote isotype-specific anti-FliC responses. Notably, TLR5, the inflammasome, and MyD88 are not absolutely required for IgG1 anti-flagellin Ab responses in mice, and FliC is capable of inducing potent IgG1 anti-FliC responses in the absence of these pathways (8). The structures on FliC that control Ab production through this unknown pathway have yet to be defined.

*Salmonella enterica* serovar Typhimurium has two flagellin genes, of which *fliC* is the phase 1 gene that is most commonly expressed and has been studied in greatest detail at the structural level (22, 23). FliCis comprised of four domains: D0, D1, D2, and D3 (22, 23). The crystallographic and cryoelectron microscopy studies of FliC and the flagellar filament have revealed that the D0 (Naip5/6) and D1 (TLR5) domains are buried deep within the filament of polymeric flagellin (22, 23). The Naip5/6 recognition site of FliC has been mapped to the carboxy-terminal 35 aa of the D0 domain, but Naip5/6 recognition of flagellin is also influenced by the N-terminal D0 domain (24, 25). FliC’s TLR5 recognition site has been mapped to highly conserved amino acid residues in the D1 domain, which has been confirmed by the crystal structure of flagellin with zebrafish TLR5 (6, 26, 27).

The D2 and the D3 domains (D2/D3) of FliC are largely exposed on the outer surface of the flagellar filament, and are the regions of the protein that are recognized by serotype specific Abs during natural *Salmonella* infections (28, 29). Although most studies have implicated the TLR5 recognition site of FliC as the critical component of flagellin’s adjuvancy and immunogenicity, there are additional reports that suggest the D2/D3 domain of flagellin is a contributing factor (30–33). Thus, there is precedence for anti-flagellin Ab responses being influenced by structures other than the well-characterized TLR5 (D1) and Naip5/6 (D0) recognition sites.

In this study, we set out to determine which structures on FliC influence Ab responses and adjuvancy. We selectively mutated the FliC molecule to determine which sites are contributing to Ab responses in mice. Using this panel of mutant flagellin proteins and mice with targeted deficiencies, we determined the molecular requirements for flagellin induced Ab responses. Our data demonstrate that a third pathway that promotes IgG1 anti-FliC requires the presence of all four domains on FliC and suggests that the structural conformation of monomeric flagellin independently of the known innate recognition sites interacts with the host’s immune system to enhance primary and secondary Ab responses.

## MATERIALS AND METHODS

### Creation of flagellin proteins

The FliC^ΔNaip5/6^, FliC^ΔTLR5^, FliC^ΔTLR5/Naip5/6^, FliC^ΔD3^, and FliC^ΔD0/D1^ genes were synthesized by Life Technologies. All FliC constructs were based on *S*. Typhimurium strain SL1344 *fliC* (GenBank accession number: CBW17983) encoding aa 1–495. FliC^ΔNaip5/6^ has a R495P amino acid substitution and addition LVPRGSHHHHHH at the C terminus. Protein FliC^ΔTLR5^ had amino acid substitutions at R90E, Q97A, E114R, R118E, E121R, D419R, and R422D and the addition of MLVPRGSHHHHHH at the N terminus. These mutations were designed to restructure the TLR5 interface with FliC based on the complex crystal structure (6), ablating key interactions while preserving the overall structure. DNA encoded FliC^ΔNaip5/6^, FliC^ΔTLR5^, FliC^ΔTLR5/Naip5/6^, FliC^ΔD3^, and FliC^ΔD0/D1^, (GenScript, Piscataway, NJ) and transformed into flagellin negative BL21 *Escherichia coli* cells for expression (Invitrogen, Life Technologies, Thermo Fisher Scientific, Grand Island, NY).

### Purification of proteins

FliC flagellin monomers were isolated from *S*. Typhimurium strain SL1344 (Δ*flgM*); purity was verified as previously described (26, 27). OVA was purchased from Sigma-Aldrich (St. Louis, MO) and ultrafiltered (Amicon; MilliporeSigma, Billerica, MA) to reduce endotoxin. Residual endotoxin from isolated flagellin monomers and OVA (Sigma-Aldrich) was removed with polymyxin B columns (Thermo Fisher Scientific). Endotoxin levels were <1 pg/μg protein, as measured with the limulus colorimetric assay (Lonza, Basel, Switzerland). For purification of recombinant flagellin proteins, BL21 *E. coli* cells transformed with pET29b flagellin containing plasmids (FliC^ΔNaip5/6^, FliC^ΔTLR5^, FliC^ΔTLR5/Naip5/6^, FliC^ΔD3^, and FliC^ΔD0/D1^) were expanded from starter cultures in Luria Broth with kanamycin (100 mg/ml) and induced with 1 mM IPTG when OD595 reached 0.6, incubated for 4 h at 37°C, and then overnight at 16°C. Bacteria were pelleted and stored at −20°C. Pellets were resuspended in standard buffer (50 mM Tris, 500 mM NaCl, 10 mM imidazole, 0.5 mg/ml lysozyme), sonicated on ice, and clarified by centrifugation. Supernatants were tumbled with 10 ml of nickel-NTA resin (Superflow NTA; QIAGEN, Valencia, CA) for 30 min at 4°C. The resin was then rinsed twice with 10 mM imidazole and once with standard buffer plus 20 mM imidazole and eluted with standard buffer plus 250 mM imidazole. Eluates were concentrated by ultrafiltration (Amicon Ultra; MilliporeSigma) and filtered through 0.22-mm Ultrafree-MC spin columns (MilliporeSigma). Proteins were purified by preparative size exclusion chromatography on Superdex 75 16/60 columns (GE Healthcare, Dallas, TX) at room temperature (RT) in 25 mM PIPES (pH 7), 150 mM NaCl, 1 mM EDTA, and 0.02% w/w sodium azide (PNEA). Residual endotoxin was removed with a Proteospin Endotoxin Removal Maxi Kit (Norgen Biotek, ON, Canada). Endotoxin levels were <1 pg/μg protein, as measured using the limulus fluorescent assay (Lonza). All purified flagellin was characterized biochemically by Coomassie and circular dichroism, prior to mouse studies.

### NF-κB luciferase reporter assay

Stably transfected CHO K1 cells were used to detect TLR5 stimulatory activity as previously described (26, 27).

### Inflammasome activation assay

Bone marrow–derived macrophages (BMDMs) were prepared from femurs of wild-type (WT) mice or mice lacking Casp1 and 11 (Casp1/11^−/−^), as previously described (8), and cultured in RPMI 1640 supplemented with 10% FBS (Atlas Biologicals, Fort Collins, CO), 10% l-cell supernatant (CSF1 source), 2 mM l-glutamine, 100 U/ml penicillin, and 100 μg/ml streptomycin (Life Technologies, Thermo Fisher Scientific) (34–36). On day 7, BMDMs were harvested and plated in 96-well plates (10^5^ cells per well). BMDMs were primed with 10 ng/ml ultrapure LPS (List Biologicals, Campbell, CA) for 3 h to induce pro–IL-1β expression. Proteins were transfected into cells using Profect-P1 lipid-based protein delivery reagent (Targeting Systems, El Cajon, CA) as previously described (8, 15, 35). IL-1β secretion was determined by ELISA (Duoset; R&D Systems, Minneapolis, MN). All assays were performed in triplicate and each experiment was repeated at least twice.

### Mice and immunizations

The University of Washington Institutional Animal Care and Use Committees approved all animal protocols. Mice were bred and housed in a specific pathogen-free facility at the University of Washington. C57BL/6 animals were purchased from Jackson Laboratories and bred in-house. MyD88^−/−^ and TLR5^−/−^xCasp1/11^−/−^ mice were bred in our animal facility (8, 37). Eight- to fourteen-week-old sex- and age-matched animals were used in all experiments. Retro-orbital bleeds were performed on all animals prior to immunization to obtain naive serum. Mice received two sequential i.p. immunizations with 30 μg flagellin and 30 μg OVA separated by 21 d. Blood was drawn 2 wk following each immunization.

### Cytokine analysis

Mouse sera were evaluated for cytokine responses at 2 and 4 h following i.p. injections with 30 μg of flagellin protein using commercially sourced IL-12/23p40 ELISA kit according to manufacturer’s instructions (Duoset; R&D Systems). IL-18 cytokine analysis was determined by ELISA, using anti-mouse IL-18 (Clone 74; R&D Systems) as a capture Ab and biotinylated anti-mouse IL-18 (Clone 93–10C; R&D Systems) as a detection Ab, as previously described (8).

### Ab analysis

High binding capacity 96-well plates (COSTAR; Corning, Amsterdam, the Netherlands) were coated with 1 μg/ml monomeric flagellin (same protein as used for immunization, unless stated otherwise) or OVA diluted in PBS (OmniPur; MilliporeSigma) and allowed to incubate overnight at RT. Plates were washed three times with PBS containing 0.05% Tween 20 and blocked for 1 h RT in PBS containing 1% BSA (Sigma-Aldrich). Plates were washed, and serial dilutions of serum were added to the wells and incubated for 1 h at RT. Plates were washed again, and HRP-conjugated secondary Abs (anti-IgG1, -IgG2c–HRP [Jackson Immunoresearch, West Grove, PA], or –IgA-HRP [BioLegend, San Diego, CA]) were added and incubated for another hour at RT. Plates were developed with TMB substrate (Thermo Fisher Scientific) and stopped with H_2_SO_4_, and absorbance was read at 450 nm (Molecular Devices, Sunnyvale, CA). Ab endpoints are presented as reciprocal log titers of the maximal serum dilution that exceeded three times the SD above the mean background absorbance.

### Statistical analysis

Significance was determined by one-way ANOVA with multiple comparison posttest, unpaired Student *t* test, or Mann–Whitney *U* test, using GraphPad Prism 5 software (La Jolla, CA). Differences were noted as significant for *p* values < 0.05.

## RESULTS

### Characterization of the mutant flagellin proteins

**FIGURE 1.**
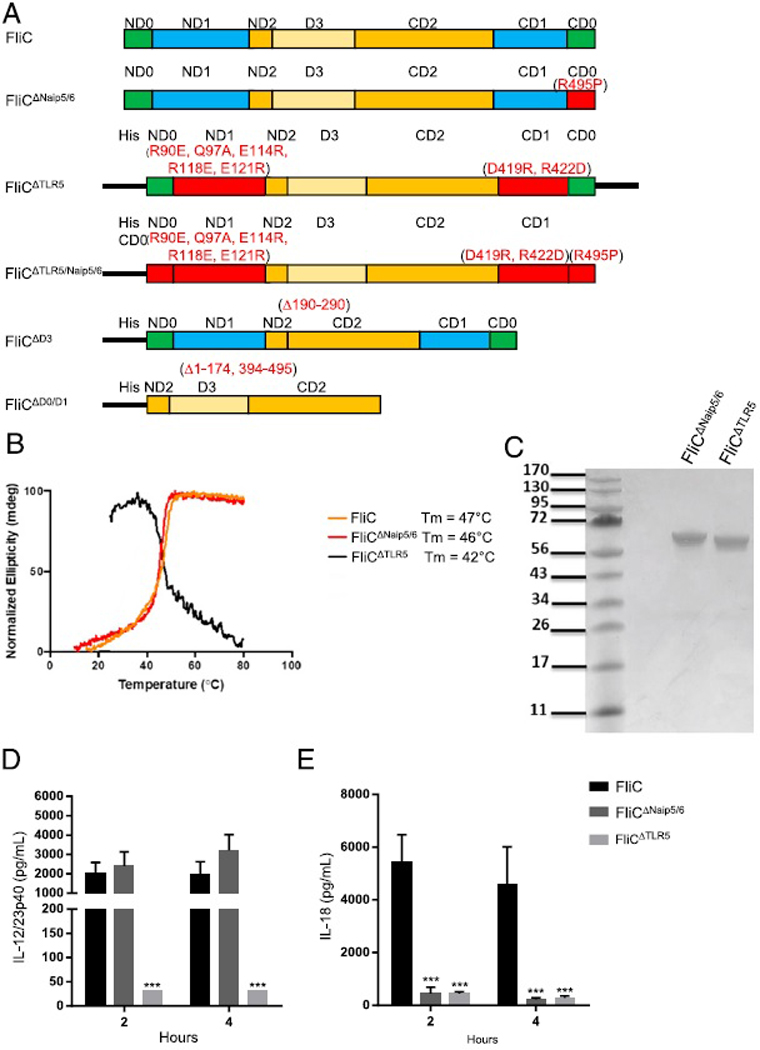
Characterization of mutant flagellin proteins. Diagram of flagellin proteins used in this study (**A**). Circular dichroism melting curves are shown for the proteins. Melting temperature (Tm) values were determined as the inflection point of the sigmoidal melting curve (**B**). Coomassie-stained SDS-PAGE gel of FliC^ΔNaip5/6^ and FliC^ΔTLR5^ used in this study (**C**). WT mice were injected i.p. with 30 μg of FliC (*n* = 5), FliC^ΔNaip5/6^ (*n* = 5), or FliC^ΔTLR5^ (*n* = 5). Serum was collected 2 and 4 h after injections and cytokine levels were determined by ELISA. IL-12/23p40 (**D**) and IL-18 (**E**) statistical analysis was done using one-way ANOVA with Bonferroni multiple comparisons posttest. ***p < 0.001.

Flagellin has been well described as a TLR5 and Naip5/6 agonist that promotes robust humoral immunity in mice (8, 9, 38). In addition, we have previously shown that TLR5-, Casp1/11-, and MyD88-independent factors also promote isotype-specific Ab responses toward *S*. Typhimurium FliC. To determine which structural features of flagellin modulate Ab production, we designed five variant FliC proteins that have ablated either Naip5/6 (FliC^ΔNaip5/6^) or TLR5 (FliC^ΔTLR5^) alone or both TLR5 and Naip5/6 recognition sites (FliC^ΔTLR5/Naip5/6^), or deleted the D3 domain (FliC^ΔD3^) and both the D0 and D1 domains (FliC^ΔD0/D1^) (Fig. 1A). Biochemical analyses of proteins indicate that they were the appropriate m.w. and properly folded (Fig. 1B, 1C).

The TLR5 stimulatory activity of FliC^ΔNaip5/6^ (EC_50_ = 0.38 ng/ml) was comparable to FliC isolated from *S*. Typhimurium (EC_50_ = 1.1 ng/ml), whereas the FliC^ΔTLR5^ variant had significantly diminished TLR5 stimulatory activity (EC_50_ = 290 ng/ml, *p* < 0.01) (Table I). We next tested the proteins for inflammasome activation. As anticipated, the FliC^ΔNaip5/6^ variant, which is mutated in the ultimate amino acid residue (R495P) and has a C-terminal 6X-His tag, was a poor inducer of IL-1β production, even at high doses (Table I, data not shown). Surprisingly, all recombinant FliC proteins (FliC^ΔTLR5^, FliC^ΔTLR5/Naip5/6^, FliC^ΔD3^, and FliC^ΔD0/D1^) also failed to induce IL-1β production when compared with equivalent concentrations of FliC (Table I). None of the proteins induced IL-1β production in Casp1/11^−/−^ BMDMs (data not shown). Thus, in vitro characterization of our flagellin proteins demonstrated that the FliC^ΔTLR5^ and FliC^ΔNaip5/6^ mutations selectively abrogated TLR5 and Naip5/6 recognition, and all the recombinant proteins had diminished inflammasome activation (8, 9, 38). Recently, it has been observed by cryoelectron microscopy that the flagellin N and C termini have multiple contact points with Naip5, suggesting the addition of 6X-His tags may interfere with Naip5 recognition (39).

Because the in vivo biological activity of our flagellin molecules may be influenced by factors that are not present in vitro, we tested the ability of our mutant proteins to induce cytokine production in in vivo. WT C57BL/6 mice were injected i.p. with 30 μg of the individual proteins; sera were collected at 2 and 4 h postinjection and tested for cytokine production. Mice that received FliC or FliC^ΔNaip5/6^ had robust production of IL-12/23p40 at both 2 and 4 h postinjection (Fig. 1D). Mice injected with FliC^ΔTLR5^ had no detectable IL-12/23p40 at either time point (Fig. 1D). As predicted from our in vitro assays, i.p. injections with FliC^ΔNaip5/6^ or FliC^ΔTLR5^ induced significantly less IL-18 when compared with mice that received FliC injections, at both the 2 and 4 h time points (Fig. 1E). Thus, in vivo characterization of the flagellin proteins agrees with our in vitro assessment of their biological activity.

### Robust IgG1 anti-flagellin responses are partially dependent on both conserved recognition sites and hypervariable domains of FliC

**FIGURE 2.**
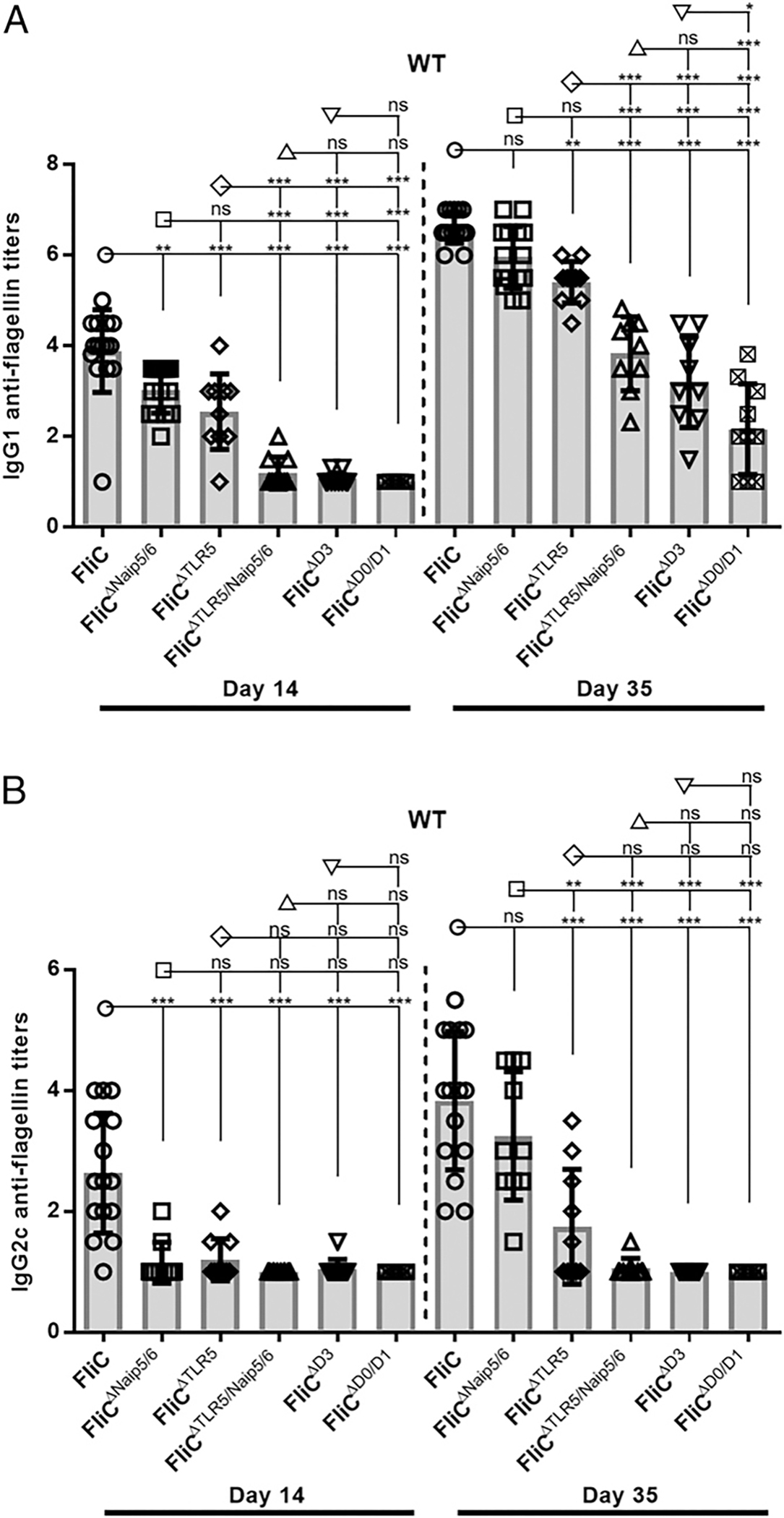
Robust IgG1 anti-flagellin responses require both conserved recognition sites and hypervariable regions of FliC. WT mice were immunized with 30 μg FliC (*n* = 15), FliC^ΔNaip5/6^ (*n* = 15), FliC^ΔTLR5^ (*n* = 10), FliC^ΔTLR5/Naip5/6^ (*n* = 11), FliC^ΔD3^ (*n* = 10), or FliC^ΔD0/D1^ (*n* = 11) on day 0 and 21, and sera were collected on days 14 and 35. Day 14 and 35 sera were analyzed for IgG1 (**A**) and IgG2c (**B**) Ab responses against FliC, FliC^ΔNaip5/6^, FliC^ΔTLR5^, FliC^ΔTLR5/Naip5/6^ FliC^ΔD3^, or FliC^ΔD0/D1^ performed by ELISA. Statistical analyses were done on day 14 and 35 using one-way ANOVA with multiple comparison posttest. *p < 0.05, **p < 0.01, ***p < 0.001. ns, not significant.

Flagellin’s immunogenicity and adjuvancy have been largely attributed to conserved sites in the D0 and D1 domains that are recognized by innate immune receptors Naip5/6 and TLR5, respectively (40). Although these innate immune receptors contribute to flagellin-dependent Ab responses, additional factors are also critical (8). To address the possibility that only the conserved TLR5 and Naip5/6 recognition sites of D0 and D1 were required for Ab responses, we immunized WT mice with our FliC^ΔTLR5^, FliC^ΔNaip5/6^, and FliC^ΔTLR5/Naip5/6^ proteins. Mice were immunized i.p. twice with 30 μg of recombinant protein on day 0 and 21, and blood was drawn 2 wk following each immunization to assess IgG1 and IgG2c titers. Primary (day 14) anti-flagellin IgG1 responses with FliC^ΔTLR5^, FliC^ΔNaip5/6^, and FliC^ΔTLR5/Naip5/6^ were significantly lower than in FliC immunized mice (Fig. 2A). Additionally, no differences were observed between FliC^ΔTLR5^ and FliC^ΔNaip5/6^ primary responses, but both were significantly higher than FliC^ΔTLR5/Naip5/6^ (Fig. 2A). In the secondary (day 35) anti-flagellin IgG1 responses, we observed no differences between FliC and FliC^ΔNaip5/6^ responses, but there was a significant reduction in mice immunized with FliC^ΔTLR5^ and an even greater reduction in secondary IgG1 titers in those WT animals that received FliC^ΔTLR5/Naip5/6^ (a median titer of 3 × 10^5^, compared with 1 × 10^4^, respectively, Fig. 2A).

To determine if flagellin’s hypervariable nonimmunogenic domain, D3, or the immunogenic D0/D1 domains were required for robust IgG1 anti-flagellin responses, we next immunized WT mice with FliC^ΔD3^ or FliC^ΔD0/D1^. Primary IgG1 anti-flagellin titers from WT mice immunized with FliC^ΔD3^ or FliC^ΔD0/D1^ were both significantly lower than in mice immunized with FliC, FliC^ΔTLR5^, or FliC^ΔNaip5/6^ (Fig. 2A). Following secondary immunization with FliC^ΔD3^ or FliC^ΔD0/D1^, anti-flagellin IgG1 Ab responses were detectable but significantly reduced relative to FliC (~1000- and 30,000-fold reduction in median titers for FliC^ΔD3^ or FliC^ΔD0/D1^, respectively; Fig. 2A).

As anticipated, IgG2c isotype-specific responses were significantly reduced following primary and secondary immunizations with FliC^ΔNaip5/6^, FliC^ΔTLR5^, and FliC^ΔTLR5/Naip5/6^ compared with FliC (Fig. 2B). Similar to anti-flagellin IgG1 primary responses, anti-flagellin IgG2c primary responses toward FliC^ΔD3^ or FliC^ΔD0/D1^ were significantly reduced compared with FliC, and following secondary immunizations, the anti-flagellin IgG2c titers remained significantly lower than responses from FliC immunized mice (~1000-fold reduction in median titers for FliC^ΔD3^ and FliC^ΔD0/D1^, respectively; Fig. 2B). Hence, our data support our previous studies that anti-flagellin IgG2c responses are dependent on the highly conserved recognition sites TLR5 and Naip5/6 and demonstrates that the hypervariable domain D3 is also required for anti-FliC IgG2c production, whereas robust IgG1 responses rely on both the known innate recognition sites as well as the hypervariable domain D3.

### MyD88-independent anti-flagellin IgG1 Ab responses do not require flagellin’s TLR5 or Naip5/6 recognition sites

**FIGURE 3.**
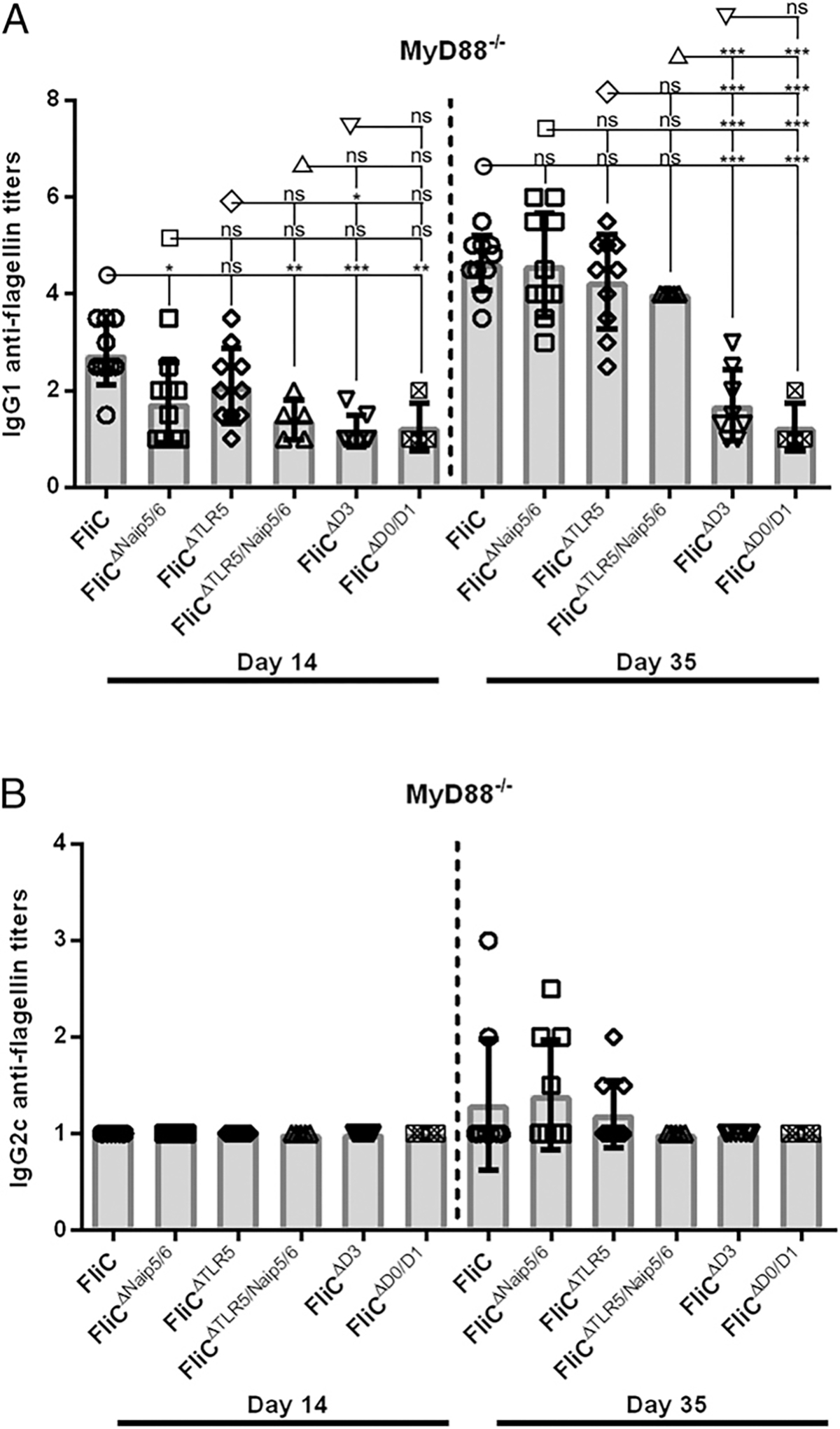
MyD88-independent IgG1 anti-flagellin responses are independent of the TLR5 and Naip5/6 recognition sites. MyD88^−/−^ mice were immunized with 30 μg WT FliC (*n* = 10), FliC^ΔNaip5/6^ (*n* = 10), or FliC^ΔTLR5^ (*n* = 10), FliC^ΔTLR5/Naip5/6^ (*n* = 5) FliC^ΔD3^ (*n* = 8), or FliC^ΔD0/D1^ (*n* = 4) on day 0 and 21, and sera were collected on days 14 and 35. Day 14 and 35 sera were analyzed for IgG1 (**A**) and IgG2c (**B**) Ab responses against WT FliC, FliC^ΔNaip5/6^, FliC^ΔTLR5^, FliC^ΔTLR5/Naip5/6^ FliC^ΔD3^, or FliC^ΔD0/D1^ performed by ELISA. Statistical analyses were done on day 14 and 35 using one-way ANOVA with multiple comparison posttest. *p < 0.05, **p < 0.01, ***p < 0.001. ns, not significant.

We previously demonstrated that anti-flagellin IgG2c responses are largely MyD88 dependent, but a substantial proportion of the anti-flagellin IgG1 response proceeds through an undefined MyD88-independent pathway (8). To further characterize this pathway, we immunized MyD88^−/−^ mice with FliC, FliC^ΔNaip5/6^, FliC^ΔTLR5^, and FliC^ΔTLR5/Naip5/6^ to determine how structural features of the innate recognition sites of flagellin contribute to the production of anti-flagellin IgG1 in the absence of MyD88. In contrast to WT mice (Fig. 2), MyD88^−/−^ mice immunized with FliC^ΔNaip5/6^ and FliC^ΔTLR5/Naip5/6^ showed significant reduction in median anti-flagellin IgG1 titers on day 14 (6–10-fold), but no significant differences were observed in secondary responses (day 35) compared with FliC immunized controls (Fig. 3A). In contrast, mice immunized with FliC^ΔTLR5^ did not differ significantly from mice immunized with FliC at either time point (Fig. 3A).

Because it appeared that neither the Naip5/6 nor the TLR5 recognition sites were essential for the induction of MyD88-independent IgG1 anti-flagellin responses, we next sought to determine if the hypervariable domain D3 played a significant role. To test the role of the D3 along with the D0/D1 domains, we immunized MyD88^−/−^ mice with FliC^ΔD3^ or FliC^ΔD0/D1^. Similar to WT immunized mice (Fig. 2), FliC^ΔD3^ or FliC^ΔD0/D1^ immunized MyD88^−/−^ mice generated significantly less primary anti-flagellin IgG1 responses when compared with FliC but did not differ much from responses of FliC^ΔNaip5/6^, FliC^ΔTLR5^, or FliC^ΔTLR5/Naip5/6^ immunized mice (Fig. 3A). Secondary immunization of MyD88^−/−^ mice with FliC^ΔD3^ or FliC^ΔD0/D1^ resulted in significantly reduced anti-flagellin IgG1 Ab responses compared with FliC, FliC^ΔNaip5/6^, FliC^ΔTLR5^, and FliC^ΔTLR5/Naip5/6^ immunized MyD88^−/−^ mice (Fig. 3A). Mice immunized with FliC^ΔD3^ or FliC^ΔD0/D1^ produced low, but detectable, anti-flagellin IgG1 secondary responses (median titers of 25 and 10, respectively compared with a median titer of 4.5 × 10^4^ for FliC; Fig. 3A).

As expected, flagellin-immunized MyD88^−/−^ mice had no detectable IgG2c following primary and low levels after secondary immunizations with all of the flagellin proteins (Fig. 3B). Our results from MyD88^−/−^ immunizations demonstrate that in addition to primary anti-flagellin IgG1 responses, secondary anti-flagellin IgG1 responses are largely MyD88 dependent in the absence of the D3 or the D0/D1 domains.

### TLR5- and Casp1/11-independent anti-flagellin Ab responses are partially dependent on the D2 and D3 domains

**FIGURE 4.**
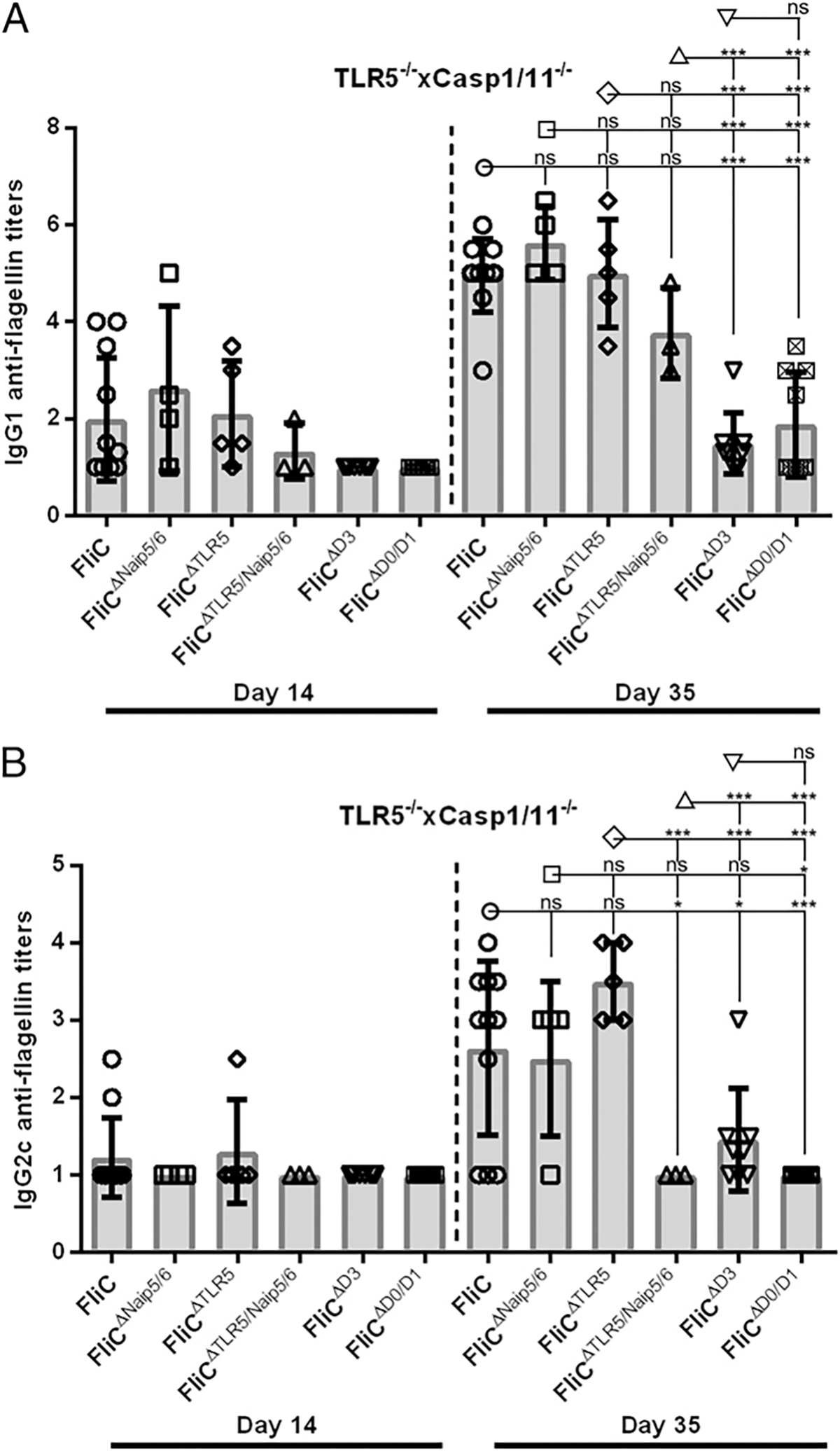
In the absence of TLR5 and inflammasome recognition, FliC’s D2/D3 domains are essential for secondary IgG1 and IgG2c anti-flagellin responses. TLR5^−/−^xCasp1/11^−/−^ mice were immunized with 30 μg WT FliC (*n* = 11), FliC^ΔNaip5/6^ (*n* = 4), or FliC^ΔTLR5^ (*n* = 5), FliC^ΔTLR5/Naip5/6^ (*n* = 3), FliC^ΔD3^ (*n* = 8), or FliC^ΔD0/D1^ (*n* = 10) on day 0 and 21, and sera were collected on days 14 and 35. Day 14 and 35 sera were analyzed for IgG1 (**A**) and IgG2c (**B**) Ab responses against WT FliC, FliC^ΔNaip5/6^ or FliC^ΔTLR5^, FliC^ΔTLR5/Naip5/6^ FliC^ΔD3^, or FliC^ΔD0/D1^ by ELISA. Statistical analyses were done on day 14 and 35 using one-way ANOVA with multiple comparison posttest. *p < 0.05, ***p < 0.001. ns, not significant.

To determine whether TLR5 and inflammasome recognition of flagellin contributes to the MyD88-independent anti-flagellin IgG1 secondary responses, we immunized mice lacking TLR5 and Casp1/11 (TLR5^−/−^xCasp1/11^−/−^) with FliC^ΔNaip5/6^, FliC^ΔTLR5^, and FliC^ΔTLR5/Naip5/6^ as described above. After primary and secondary immunizations, FliC, FliC^ΔNaip5/6^, FliC^ΔTLR5^, and FliC^ΔTLR5/Naip5/6^ immunized mice did not significantly differ in anti-flagellin IgG1 responses (Fig. 4A).

Next, to determine if the hypervariable domains of flagellin were critical for TLR5- and Casp1/11-independent anti-flagellin responses, we proceeded to immunize TLR5^−/−^xCasp1/11^−/−^ mice with FliC^ΔD3^ or FliC^ΔD0/D1^. Primary IgG1 responses were undetectable in all TLR5^−/−^xCasp1/11^−/−^ mice that received FliC^ΔD3^ or FliC^ΔD0/D1^ (Fig. 4A). Secondary immunizations yielded significantly reduced IgG1 titers for FliC^ΔD3^ (median titer 20) and FliC^ΔD0/D1^ (median titer 10) compared with FliC (median titer 1 × 10^5^) (Fig. 4A). Furthermore, secondary immunizations with FliC^ΔD3^ or FliC^ΔD0/D1^ also led to significantly reduced anti-flagellin IgG1 titers when compared with TLR5^−/−^xCasp1/11^−/−^ mice immunized with FliC, FliC^ΔNaip5/6^, FliC^ΔTLR5^ or FliC^ΔTLR5/Naip5/6^ (Fig. 4A).

The primary IgG2c responses in the TLR5^−/−^xCasp1/11^−/−^ mice displayed median titers that were below our limit of detection (Fig. 4B). Secondary anti-flagellin IgG2c responses showed no difference between FliC, FliC^ΔNaip5/6^ or FliC^ΔTLR5^, but FliC^ΔTLR5/Naip5/6^ displayed significantly lower secondary IgG2c responses than FliC immunized TLR5^−/−^xCasp1/11^−/−^ controls (Fig. 4B). Secondary immunizations with FliC^ΔD0/D1^ induced no detectable anti-flagellin IgG2c responses, and FliC^ΔD3^ induced significantly reduced IgG2c secondary responses in TLR5^−/−^xCasp1/11^−/−^ mice (median titer of 20) compared with FliC (median titer 1 × 10^3^) (Fig. 4B). Therefore, our data suggest TLR5- and Casp1/11-independent Ab responses require either FliC’s D0/D1/D2 or D2/D3 domains.

### FliC-dependent adjuvancy toward OVA relies on the D0 and D1 domains

**FIGURE 5.**
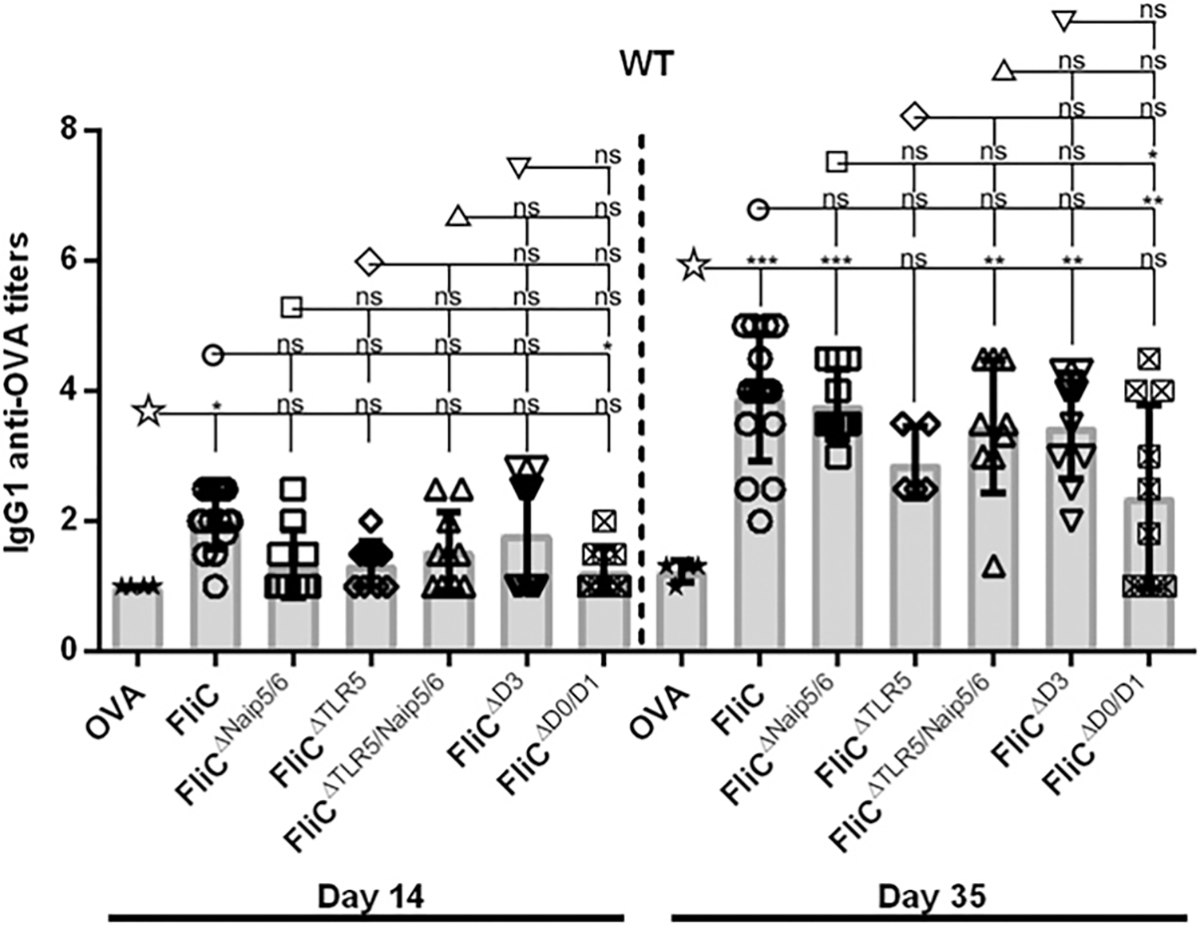
Robust flagellin-mediated IgG1 anti-OVA responses require FliC’s D0/D1 domains. WT mice given WT FliC (*n* = 15), FliC^ΔNaip5/6^ (*n* = 10), FliC^ΔTLR5^ (*n* = 10), FliC^ΔTLR5/Naip5/6^ (*n* = 11), FliC^ΔD3^ (*n* = 11), or FliC^ΔD0/D1^ (*n* = 11) were immunized twice on day 0 and 21 with 30 μg flagellin and 30 μg OVA, and sera were collected on days 14 and 35. Day 14 and 35 sera were analyzed for IgG1 specific Ab responses against OVA by ELISA. Statistical analyses were done on day 14 and 35 using one-way ANOVA with multiple comparison posttest. *p < 0.05, **p < 0.01, ***p < 0.001. ns, not significant.

Flagellin has also been demonstrated to be a potent vaccine adjuvant toward nonimmunogenic Ags that are not physically linked to FliC that induces predominantly IgG1 Ab responses against the Ag. Similar to anti-flagellin Ab responses, Ab responses directed against model Ags coadministered with flagellin are partially dependent on TLR5, Casp1, and MyD88 (8). WT mice were immunized with FliC^ΔNaip5/6^, FliC^ΔTLR5^, FliC^ΔNaip5/6/TLR5^, FliC^ΔD3^, or FliC^ΔD0/D1^ and were coadministered with 30 μg of OVA i.p. on day 0 and 21. WT mice that were immunized with FliC^ΔNaip5/6^, FliC^ΔTLR5^, FliC^ΔTLR5/Naip5/6^, or FliC^ΔD3^ and OVA did not demonstrate any significant reduction in their primary or secondary IgG1 anti-OVA response compared with FliC plus OVA (Fig. 5). However, mice immunized with FliC^ΔD0/D1^ and OVA had significantly reduced primary and secondary IgG1 anti-OVA responses compared with controls given FliC and OVA (Fig. 5). Furthermore, as predicted, we observed no significant differences in IgG1 anti-OVA responses between FliC and any of the mutant proteins in TLR5^−/−^xCasp1/11^−/−^ mice were seen (data not shown). Hence, our results demonstrate that the FliC’s extrinsic adjuvancy against nonimmunogenic Ags relies on the presence of the D0 and D1 domains.

### FliC-induced IgA responses are dependent on the TLR5 recognition site

**FIGURE 6.**
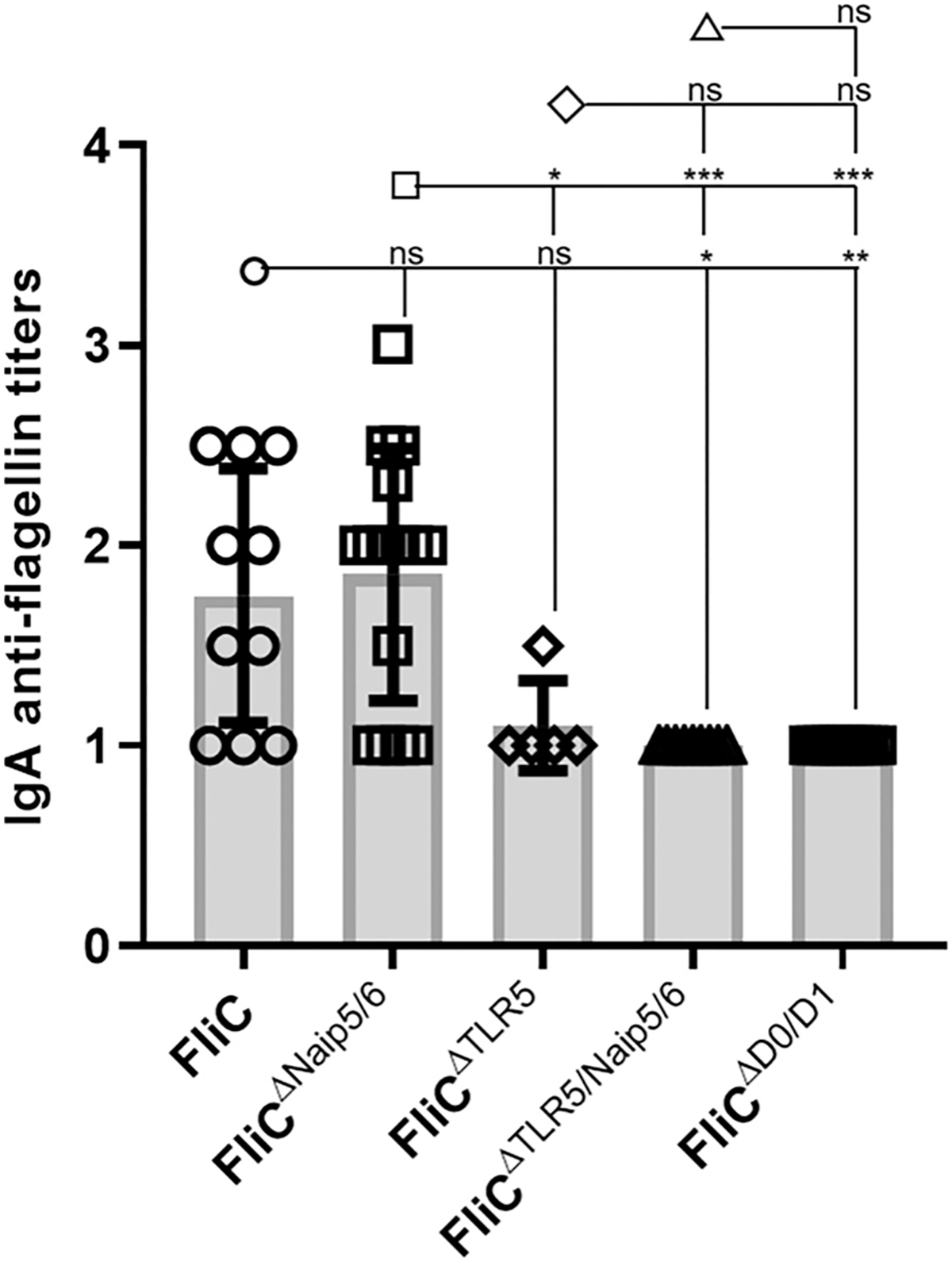
IgA anti-FliC responses are dependent on the TLR5 recognition site. WT mice given WT FliC (*n* = 10), FliC^ΔNaip5/6^ (*n* = 15), FliC^ΔTLR5^ (*n* = 5), FliC^ΔTLR5/Naip5/6^ (9), or FliC^ΔD0/D1^ (*n* = 5) were immunized twice on day 0 and 21 with flagellin, and sera were collected on day 35. Naive and day 35 sera were analyzed for IgA specific Ab responses against WT FliC, FliC^ΔNaip5/6^, FliC^ΔTLR5^, FliC^ΔTLR5/Naip5/6^, or FliC^ΔD0/D1^ by ELISA. Statistical analyses were done on day 35 using one-way ANOVA with multiple comparison posttest. *p < 0.05, **p < 0.01, ***p < 0.001. ns, not significant.

Flagellin induces low titer IgA Abs through a TLR5- and MyD88-dependent pathway (8, 21). Using our flagellin proteins, we determined which structures were required to induce IgA anti-flagellin Abs. Anti-FliCIgA responses were detectable following secondary immunizations in mice immunized with FliC and FliC^ΔNaip5/6^ protein but not FliC^ΔTLR5^, FliC^ΔTLR5/Naip5/6^, or FliC^ΔD0/D1^ (Fig. 6). Hence, these data agree with our previous report demonstrating that anti-flagellin IgA responses are strictly dependent on the conserved TLR5 recognition site.

## DISCUSSION

Previously, we described how innate flagellin receptors, TLR5 and Naip5/6, work in concert to promote IgG1 and IgG2c anti-FliC responses (8). Although anti-flagellin IgG2c responses are largely dependent on TLR5, Casp1, and MyD88, the anti-flagellin and anti-OVA IgG1 responses were only partially dependent on these host molecules, revealing a TLR5-, inflammasome-, and MyD88-independent pathway used by flagellin to induce Ab responses (8). In this article, we provide a more detailed understanding of this pathway by examining the structural components of bacterial flagellin that contribute to Ab responses in mice. Consistent with our previous studies in knockout mice, WT mice immunized with FliC^ΔNaip5/6^ or FliC^ΔTLR5^ displayed significant reductions in IgG2c (T_H_1) responses, and animals receiving FliC^ΔTLR5/Naip5/6^ had no IgG2c responses, confirming that T_H_1 type Ab responses against flagellin are influenced by both TLR5 and the Naip5/6 inflammasome (8). These results support our previous conclusion that flagellin recognition through TLR5 and Naip5/6 work in concert to promote robust IgG2c responses (8).

Our results from WT mice immunized with FliC^ΔNaip5/6^, FliC^ΔTLR5^, or FliC^ΔTLR5/Naip5/6^ also support our conclusion that TLR5 and Naip5/6 play redundant roles in generating anti-flagellin IgG1 responses (8). Because anti-flagellin IgG1 Abs are the dominant isotype produced in flagellin-immunized WT mice, these results are also consistent with the previous conclusions of Vijay-Kumar and colleagues (38) that either TLR5- or Nlrc4-mediated recognition of FliC is sufficient for robust anti-flagellin IgG responses (8). No significant differences were observed in secondary IgG1 anti-FliC titers from MyD88^−/−^ and TLR5^−/−^xCasp1/11^−/−^ mice immunized with FliC, FliC^ΔNaip5/6^, FliC^ΔTLR5^, or FliC^ΔTLR5/Naip5/6^, demonstrating that the third unknown pathway that promotes IgG1 responses against FliC does not require the conserved TLR5 or the Naip5/6 recognition sites on flagellin to induce strong secondary responses against flagellin (Figs. 3, 4).

As expected, MyD88^−/−^ mice immunized with FliC^ΔD3^ or FliC^ΔD0/D1^ were unable to generate IgG2c responses (Fig. 3B). In contrast to MyD88^−/−^ mice immunized with FliC, mice that received either FliC^ΔD3^ or FliC^ΔD0/D1^ had significantly reduced IgG1 titers, which was also observed in TLR5^−/−^xCasp1/11^−/−^ mice (Figs. 3, 4). We believe that the TLR5-, inflammasome-, and MyD88-independent pathway that promotes anti-flagellin responses requires the naturally occurring structure of *S*. Typhimurium flagellin and that in the absence of any one of these domains in combination with the genetic ablation of innate receptors, IgG1 directed against FliC is significantly reduced.

Results from WT mice immunized with FliC^ΔD3^ or FliC^ΔD0/D1^ indicate that primary anti-flagellin responses are highly dependent on FliC’s D0, D1, D2, and D3 domains (Fig. 2). We conclude that potent primary anti-flagellin responses requires the preservation of all four domains, regardless of their immunogenic properties. Our results are in alignment with previous reports, which also concluded that the D2/D3 domain contributes to the immunogenicity of *Salmonella*’*s* flagellin (30–32). In addition, mice immunized with FliC make Ab responses that show reactivity to the D0/D1 and D2/D3 domains (Supplemental Fig. 1), indicating that both components of the molecule are antigenic and that there is not a hole in the mouse BCR repertoire for any of FliC’s domains. This is consistent with previously published work from Sanders and colleagues (10), who concluded that Abs generated against monomeric flagellin are capable of recognizing the entire surface of the protein, whereas Abs generated against polymerized flagella are primarily directed against the D2/D3 domains, the major exposed surface in the filament that is available for Ab binding. Thus, we postulate that the structural conformation of all four domains of FliC are required to engage the TLR5-, Naip5/6-, and MyD88-independent pathway to enhance robust primary Ab responses that are also augmented by innate detection of flagellin through TLR5 and the inflammasome. Currently, the nature of this pathway is poorly understood and requires further investigation. Possible interactions include novel flagellin receptors and natural immunity.

A major unresolved question is the mechanism responsible for innate recognition-independent enhancement of anti-flagellin Ab responses. Understanding how FliC’s immunogenic and nonimmunogenic domains enhance anti-flagellin Ab production is an important aim for future investigations. Sequence alignment of FliC with flagellin molecules from *Listeria monocytogenes* and *Salmonella* adelaide reveals that the majority of the homology between the flagellin molecules resides within the D0 and D1 domains and a limited sequence of D2. Moreover, the D3 domain is hypervariable and only shares homology with a small subset of *S.* Typhimurium with identical serotype (data not shown). Thus, it remains unclear what amino acid regions from the hypervariable domains mitigate this third unknown pathway and whether this attribute is shared by other flagellin molecules or limited to close relatives of FliC. Nempont and colleagues (30) described three different FliC deletions Δ204–292, Δ191–352, and Δ174–400 that had reduced immunogenicity, although the most severely attenuated mutants were the Δ191–352 and Δ174–400 deletions, suggesting that the D2 domain may be the most critical for MyD88-independent IgG1 anti-flagellin responses (33, 41). Future studies using flagellin from *L*. *monocytogenes* and *S*. *adelaide* to define the precise structures on D2/D3 that are required to enhance Ab production will be helpful to understand FliC’s biological activity and also for vaccine development.

Because the D2/D3 has very limited homology with other flagellin molecules, it seems unlikely that a dedicated innate immune receptor recognizes FliC’s D2/D3 domain and is responsible for inducing robust primary anti-flagellin Ab responses. One possible mechanism is suggested by results observed over four decades ago by Nossal, Ada, and colleagues (42, 43). Their studies with *Salmonella adelaide* flagellin and flagella demonstrated that flagellin is targeted to lymphoid follicles in rats and that a cyanogen bromide fragment of flagellin, peptide A, which roughly corresponds to the D2/D3 domain (data not shown), was also targeted to lymphoid follicles (42, 43). Furthermore, they demonstrated that the targeting of flagella and flagellin to lymphoid follicles was influenced by microbiota and radiosensitive cells, suggesting that microbiota-dependent natural Abs may be responsible for targeting flagellin to lymphoid follicles (44). Another possibility is suggested by the adhesive properties that have been attributed to flagella from various bacteria. *Salmonella* flagella and FliC bind cholesterol and are needed for biofilm formation (45). *Salmonella* and *Pseudomonas* flagellins have also been demonstrated to bind gangliosides (46). Thus, it is possible that FliC interacts with host cell surface molecules and that this interaction enhances primary Ab responses, possibly through preferential targeting of flagellin to appropriate targets within lymph nodes.

The rationale design of effective vaccines to meet the clinical needs to combat numerous infectious diseases such as influenza, malaria, and HIV, and emerging diseases such as Ebola and Zika, requires in depth knowledge of the molecular mechanisms that enhance the efficacy of immunogens. There are ample mouse model data that support the role of flagellin’s TLR5 and Naip5/6 stimulatory activity in promoting immune responses, and now we provide further evidence for an additional third pathway that also promotes FliC’s robust immunogenicity. Understanding this third pathway and if it is conserved in humans is significant because human NAIP does not recognize flagellin, and multiple ongoing clinical trials are using flagellin as an adjuvant. Furthermore, it has been reported that the addition of influenza A Ags to flagellin is capable of inducing Ab-specific responses against the influenza Ag (47). In this study, we demonstrated that all four of FliC’s domains mediate robust primary and secondary Ab responses and functions independently of the known innate immune recognition pathways for bacterial flagellin. Further characterization of the innate recognition-independent pathway will provide valuable insight into novel strategies to rationally design flagellin-based vaccines with maximum efficacy.

## Supplementary Material

Supplemental data

## Figures and Tables

**TABLE I.** TLR5 activity is independent of inflammasome recognition

Flagellin	TLR5 EC_50_ (ng/ml)	Inflammasome EC_50_ (ng/ml)

FliC	1.2	2.0
FliC^ΔNaip5/6^	0.4	ND
FliC^ΔTLR5^	290	ND
FiiC^ΔTLR5/Naip5/6^	ND	ND
FliC^ΔD3^	0.1	ND
FliC^ΔD0/D1^	ND	ND

## Abbreviations used in this article:

BMDM: bone marrow–derived macrophage
Casp1: caspase 1
NLR: NOD-like receptor
PAMP: pathogen-associated molecular pattern
PRR: pattern recognition receptor
RT: room temperature
WT: wild-type
