# Supplementary material for: FliC’s Hypervariable D3 Domain Is Required for Robust Anti-Flagellin Primary Antibody Responses
Source: Immunohorizons. Author manuscript; Available in PMC 2024 Dec 17. (PMC11650696; doi:10.4049/immunohorizons.1800061)
Supplement: Supplemental data [file NIHMS1991181-supplement-Supplemental_data.pdf]

Supplemental Figure 1

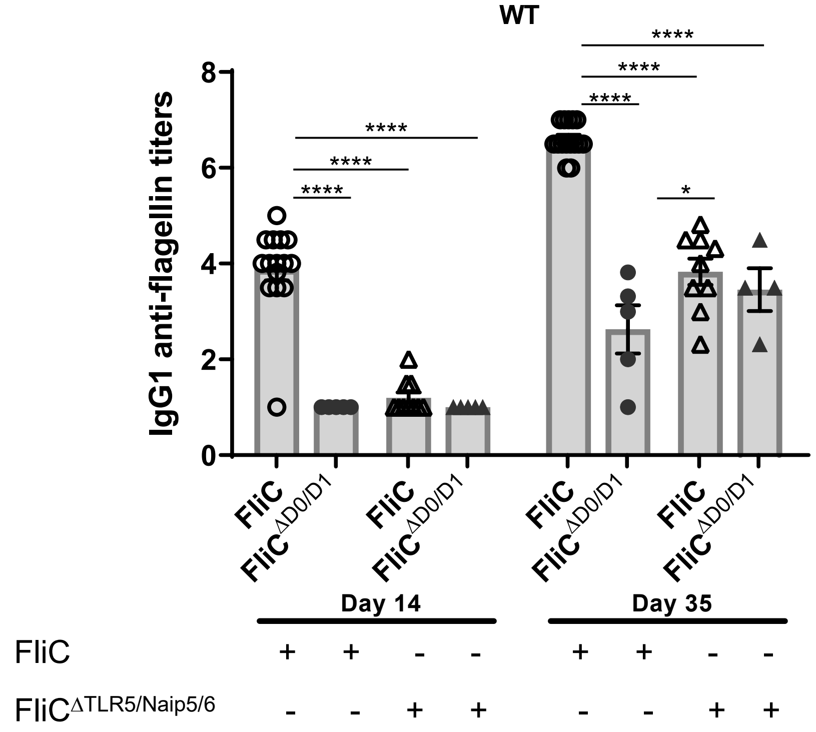

Supplemental Figure 1. FliC generates antibody responses not specific to the D2 or D3 domains of *S*. Typhimurium flagellin. WT mice given WT FliC or FliC$^{\Delta TLR5/Naip5/6}$ were immunized twice on day 0 and day 21 with flagellin, and sera collected on days 14 and 35. Days 14 and 35 sera were analyzed for IgG1 specific antibody responses against WT FliC or FliC$^{\Delta D0/D1}$ by ELISA.

Downloaded from http://journals.aai.org/immunohorizons/article-supplement/3819/pdf/ih_1800061_supplemental_figure_1/ by guest on 02 May 2024
